# Dose coverage of axillary level I-III areas during whole breast irradiation with simplified intensity modulated radiation therapy in early stage breast cancer patients

**DOI:** 10.18632/oncotarget.4301

**Published:** 2015-05-27

**Authors:** Li Zhang, Zhao-zhi Yang, Xing-xing Chen, Jeffrey Tuan, Jin-li Ma, Xin Mei, Xiao-li Yu, Zhi-rui Zhou, Zhi-min Shao, Guang-yu Liu, Xiao-mao Guo

**Affiliations:** ^1^ Department of Radiation Oncology, Fudan University Shanghai Cancer Center, Department of Oncology, Shanghai Medical College, Fudan University, Shanghai, China; ^2^ Department of Breast Surgery, Fudan University Shanghai Cancer Center, Department of Oncology, Shanghai Medical College, Fudan University, Shanghai, China; ^3^ Department of Radiation Oncology, National Cancer Centre Singapore, Singapore

**Keywords:** breast cancer, positive sentinel lymph nodes, radiotherapy, intensity modulated radiation therapy, axillary level

## Abstract

**Purpose:**

This study was designed to evaluate the dose coverage of axillary areas during whole breast irradiation with simplified intensity modulated radiation therapy (s-IMRT) and field-in-field IMRT (for-IMRT) in early stage breast cancer patients.

**Methods:**

Sixty-one consecutive patients with breast-conserving surgery and sentinel lymph node biopsy were collected. Two plans were created for each patient: the s-IMRT and for-IMRT plan. Dosimetric parameters of axillary areas were compared.

**Results:**

The average of mean doses delivered to the axillary level I areas in s-IMRT and for-IMRT plan were 27.7Gy and 29.1Gy (*p* = 0.011), respectively. The average of V47.5Gy, V45Gy and V40Gy (percent volume receiving≥ 47.5Gy, 45Gy and 40Gy) of the axillary level I in s-IMRT plan was significantly lower than that in for-IMRT plan (*p* < 0.001). For for-IMRT plans, patients with upper tangential border to humeral head ≤2cm, breast separation >19.3cm and body width >31.9cm had significantly higher mean dose in axillary level I area (*p* = 0.002, 0.007, 0.001, respectively).

**Conclusion:**

Compared with for-IMRT plan, the s-IMRT plan delivered lower dose to axillary level I area. For centers using s-IMRT technique, caution should be exercised when selecting to omit axillary lymph node dissection for patients with breast conserving surgery and limited positive SLNs.

## INTRODUCTION

For patients with breast cancer and positive sentinel lymph nodes (SLNs), the standard treatment has traditionally been complete axillary lymph node dissection (ALND) [1]. Recently, the AMAROS trial found that axillary radiotherapy could achieve excellent and comparable axillary control as ALND with only about half rate of lymphedema in women with early stage breast cancer and limited positive SLNs, suggesting that radiotherapy may be a better option than ALND [2]. However, axillary radiotherapy may lead to overtreatment. Another two trials — Z0011 and IBSCG 23-01 trials demonstrated equivalent survival between SLNB alone and SLNB followed by ALND in early stage breast cancer patients with limited positive SLNs [3, 4]. In both trials, regional recurrence with SLNB alone was only 0.9% and 0.8%, despite an estimated 27% and 13% of patients having additional metastases in the remaining axillary lymph nodes [4, 5]. One of the possible reasons for the low rate of axillary recurrence was attributed to the dose coverage to axilla during whole breast irradiation.

Whole breast irradiation is an important part of the breast conserving therapy. Along with a satisfactory regional control rate and survival benefit [6], breast radiotherapy based on tangential fields technique leads to acute skin toxicity, poor cosmetic effect and psychological morbidity due to dose inhomogeneity, especially for large-breasted patients [7, 8]. IMRT is a newer radiation technique that has been increasingly adopted as an adjuvant treatment after breast-conserving surgery. IMRT can improve breast dose homogeneity and translate into decreased radiation-related complications [9-11]. Various options for forward and inverse-optimized breast IMRT exist. In Fudan University Shanghai Cancer Center, we have clinically implemented the linac-based inverse-planned s-IMRT technique for its advantage on delivery efficiency compared to conventional IMRT [12].

Although several prior studies have evaluated the incidental dose to axilla during whole breast irradiation with tangential fields [13-19], which was the radiation technique used in Z0011, the dose coverage of axilla with the s-IMRT technique is unclear. It should be noted that the s-IMRT technique might deliver lower dose to axilla because of its more conformal dose distribution. In this study, we evaluated the dose coverage of axillary level I, II, III areas during whole breast irradiation with the s-IMRT technique and compared dosimetric parameters with the for-IMRT technique. We also analyzed the potential factors that affected dose distribution of axilla.

## RESULTS

### Patients' characteristics

Sixty-one consecutive breast cancer patients with breast-conserving surgery who were treated in Fudan University Shanghai Cancer Center were selected. Among them, 57 (93.4%) patients had negative SLNs, 4 (6.6%) patients had micrometastatic SLNs. Clinical and pathological characteristics of patients were shown in Table 1.

**Table 1:** Clinical and pathological characteristics of patients

Variable	Number (n = 61)	Percent (%)
Age (years)		
Median	50	
Range	28~77	
BMI (kg/m^2^)		
Median	21.7	
Range	18.3~29.1	
Tumor side		
Left	30	49.2
Right	31	50.8
Quadrant		
Upper quadrant	45	73.8
Lower quadrant	11	18.0
Nipple level	5	8.2
T stage		
T1	49	80.3
T2	12	19.7
SLN status		
Negative	57	93.4
Micro-metastasis	4	6.6
Hormone receptor status		
ER/PR positive	47	77.0
ER/PR negative	14	23.0
HER-2 status		
Positive	47	77.0
Negative	14	23.0

### Dose coverage of axillary level I, II, III areas in s-IMRT and for-IMRT plan

**Figure 1:**
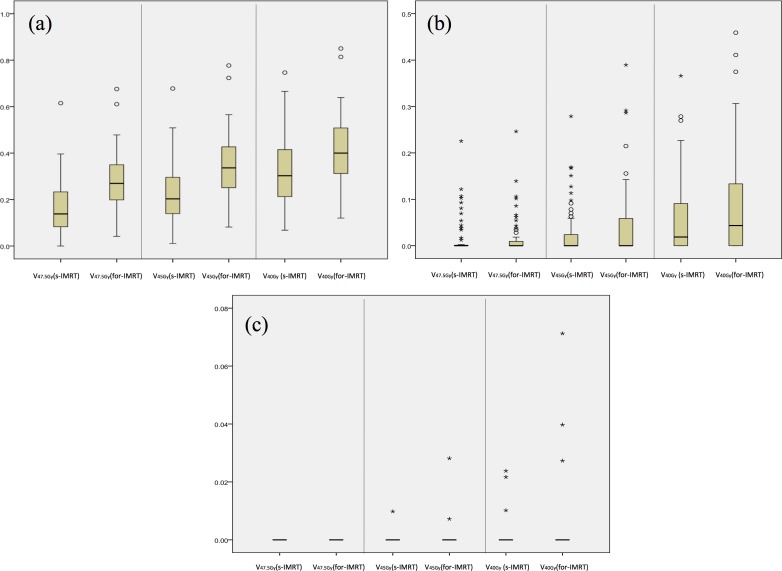
Boxplots of V47.5Gy, V45Gy, V40Gy values of the axillary level I, II, III areas **a.** Axillary level I area, **b.** axillary level II area, **c.** axillary level III area. The box includes the 25%–75% of the data. The solid line within the box is the median value. Values more than 1.5 times but less than 3 times of interquartile range from the end of the box are outliers and labeled as circles, Values more than 3 times of interquartile range from the end of the box are extremes and labeled as asterisks.

As showed in Table 2. The average of mean dose delivered to the axillary level I, II, III areas in s-IMRT and for-IMRT plan were 27.7Gy (95%CI was 26.1~29.4Gy) and 29.1Gy (95%CI was 27.2~31.0Gy) (*p* = 0.011), 10.6Gy (95%CI was 8.9~12.3Gy) and 10.9Gy(95%CI was 9.2~12.6Gy) (*p* = 0.403), 2.5Gy (95%CI was 2.1~3.1Gy) and 2.8Gy (95%CI was 2.3~3.4Gy) (*p* = 0.089), respectively. The average of V47.5Gy, V45Gy and V40Gy of the axillary level I in s-IMRT plan were significant lower than that in for-IMRT plan (all *p* < 0.001). The average of V47.5Gy, V45Gy and V40Gy of the axillary level II and III areas were all very low. The variability of the V47.5Gy, V45Gy and V40Gy in the axillary level I, II, III areas were shown in Figure 1.

**Table 2:** Dosimetric parameters of axilla and organs at risk in s-IMRT versus for-IMRT plan

Structures	Parameters	Mean (95% CI)		*P* value
		S-IMRT plan	For-IMRT plan	
**Axillary levels**				
Axillary level I	Dmean (Gy)	27.7 (26.1~29.4)	29.1 (27.2~31.0)	0.011
	V47.5Gy (%)	16.9 (14.1~20.0)	27.6 (24.6~30.7)	< 0.001
	V45Gy (%)	22.1 (19.0~25.4)	34.5 (31.0~38.1)	< 0.001
	V40Gy (%)	31.3 (28.0~35.1)	41.1 (37.3~45.0)	< 0.001
Axillary level II	Dmean (Gy)	10.6 (8.9~12.3)	10.9 (9.2~12.6)	0.403
	V47.5Gy (%)	1.7 (0.8~2.9)	1.8 (0.8~2.9)	0.909
	V45Gy (%)	2.7 (1.5~4.4)	4.4 (2.6~6.7)	0.011
	V40Gy (%)	5.7 (3.9~8.0)	8.3 (5.7~11.3)	0.002
Axillary level III	Dmean (Gy)	2.5 (2.1~3.1)	2.8 (2.3~3.4)	0.089
	V47.5Gy (%)	0.0 (0.0~0.0)	0.0 (0.0~0.0)	--
	V45Gy (%)	0.0 (0.0~0.0)	0.1 (0.0~0.2)	0.409
	V40Gy (%)	0.1 (0.0~0.2)	0.2 (0.0~0.6)	0.198
**Organs at risk**				
Ipsilateral lung	Dmean (Gy)	8.5 (8.2~8.8)	8.9 (8.5~9.2)	< 0.001
	V30Gy (%)	10.6(10.0~11.3)	13.5 (12.7~14.2)	< 0.001
	V20Gy (%)	14.7 (14.0~15.4)	16.8 (15.9~17.6)	< 0.001
Heart	Dmean (Gy)	4.1 (3.7~4.5)	4.9 (4.3~5.5)	0.001

### Impact of tangential fields' height and patients' anatomic features

In for-IMRT plan, Patients with distance from upper tangential border to the humeral head ≤2cm had a significant higher mean dose than those > 2cm (31.3Gy *vs* 25.4Gy, *p* = 0.002). Patients with breast separation > 19.3cm, body width > 31.9cm had a significant higher mean dose than those with breast separation ≤19.3cm, body width ≤31.9cm (31.7Gy *vs* 26.5Gy, *p* = 0.007; 32.4Gy *vs* 25.9Gy, *p* = 0.001). Patients with breast CTV > 415cm^3^ also had the trend of having a significant higher mean dose than those ≤415cm^3^ (30.9Gy *vs* 27.3Gy, *p* = 0.06). In s-IMRT plan, all of these parameters did not significantly impact the mean dose delivered to the axillary level I area (Table 3).

**Table 3:** Factors that impact the mean dose of axillary levels I

Parameters	Mean (95% CI)	*P* value	Mean (95% CI)	*P* value
	S-IMRT plan		For-IMRT plan	
BMI		0.291		0.354
≤22kg/m^2^	28.6 (26.2~31.1)		28.2 (25.8~30.6)	
>22kg/m^2^	26.7 (23.9~29.5)		30.0 (26.8~33.3)	
Breast CTV		0.214		0.06
≤415cm^3^	26.6 (24.2~29.0)		27.3 (25.0~30.0)	
>415cm^3^	28.9 (26.0~31.7)		30.9 (27.7~34.1)	
Breast separation		0.915		0.007
≤19.3cm	27.8 (25.3~30.4)		26.5 (24.0~29.1)	
>19.3cm	27.6 (24.9~30.4)		31.7 (28.9~34.5)	
Thoracic index		0.177		0.863
≤0.32	26.5 (23.9~29.1)		28.9 (25.9~31.9)	
>0.32	29.0 (26.4~31.5)		29.2 (26.5~31.9)	
Body width		0.872		0.001
≤31.9cm	27.9 (25.3~30.4)		25.9 (23.5~28.3)	
>31.9cm	27.6 (24.8~30.3)		32.4 (29.6~35.1)	
Body depth		0.987		
≤17.6cm	27.7 (25.3~30.1)		29.1 (27.0~31.2)	0.996
>17.6cm	27.7 (24.9~30.6)		29.1 (25.6~32.5)	
The distance from upper tangential border to humeral head		0.575		0.002
≤2cm	27.3 (25.2~29.3)		31.3 (29.3~33.3)	
>2cm	28.4 (24.7~31.8)		25.4 (21.8~29.0)	

### Radiation dose of ipsilateral lung and heart in s-IMRT and for-IMRT plan

The s-IMRT plan significantly reduced the mean dose, the average of V30Gy, V20Gy of the ipsilateral lung as compared with for-IMRT plan (all *p* < 0.001). For the heart in left-sided patients, the s-IMRT plan significantly reduced the mean dose (*p* = 0.001) (Table 2).

## DISCUSSION

This study evaluated dose coverage of axilla during whole breast irradiation with the s-IMRT technique and compared dosimetric parameters with that using the for-IMRT technique. We found that when s-IMRT or for-IMRT plan delivered dose to axillary level I, II, III areas in patients with whole breast irradiation, the s-IMRT plan reduced axillary level I area dose as compared to the for-IMRT plan.

Prior studies that analyzed dose coverage of axilla during whole breast irradiation were mainly based on 2D or 3D tangential fields. Krasin et al [14] demonstrated that whole breast irradiation using standard tangential fields did not fully cover axilla. In their study, the mean dose of axillary level I, II, III areas were 32.0Gy, 26.5Gy, 18.2Gy, respectively, only 1 out of 25 patients had adequate coverage of the level I area. Aristei et al [15] evaluated axillary dose in 35 patients using standard tangential fields, Axillary Levels I and II were delineated on CT slices on the basis of anatomic landmarks, they found that the median dose administered to level I and II were 38.58Gy and 20.65Gy, respectively. Orecchia et al [16] analyzed dose coverage of axillary level I in standard tangential fields of 15 patients treated with quadrantectomy and SLNB. The axillary level I was contoured on CT scans from the site of surgical clip up to the sternal manubrium. They reported the maximum dose mean ranged from 5% to 80% of the prescribed dose (mean value 48.7%). The mean total dose received by the volume of interest was lower than 40Gy in all but one patient. In our study, the mean dose delivered to axillary level I, II, III areas in s-IMRT and for-IMRT plans were 27.7Gy and 29.1Gy, 10.6Gy and 10.9Gy, 2.5Gy and 2.8Gy, respectively. Our results were consistent with these and other similar studies [13, 17], suggesting inadequate coverage of axillary level I, II, III areas during whole breast irradiation.

In addition, we found that the mean dose, the average of V47.5Gy, V45Gy and V40Gy of axillary level I area were all significantly lower in s-IMRT plan than that in for-IMRT plan. This might be due to the steeper dose gradient around the breast target volume in the s-IMRT plan that would include lesser axillary tissue. Another study by Kataria et al [20] reported similar result that conformal techniques (tangential-IMRT, 3D conformal radiotherapy) delivered significantly lesser incidental radiation to lower axilla than standard tangential fields.

Recently, Jagsi et al [21] reported the radiation field design used for whole breast irradiation in the Z0011 trial and showed that among 142 patients with sufficient records to evaluate tangential height, superior border of tangential fields was within 2 cm of the humeral head (high tangential fields) in approximately half of the patients in both arms. High tangential fields have been showed to deliver higher dose to axilla than standard tangential fields. Reznik et al [18] found that the mean dose delivered to axillary level I, II, III areas were 66%, 44%, 31% of the prescribed dose with standard tangential fields, respectively, and increased to 86%, 71%, and 73% with high tangential fields. Belkacemi et al [19] demonstrated an increase from a mean dose of 20Gy and 4Gy with standard tangential fields to 33Gy and 11Gy with high tangential fields in axillary level I and II areas. The present study had similar result, suggesting that it might be safer to treat patients with high tangential fields than standard tangential fields when omitting further ALND in the event that SLN was positive.

Except for the height of tangential fields, our study demonstrated that anatomic features of patients impacted the dose distribution of axillary level I area with breast separation > 19.3cm, body width > 31.9cm received higher dose to axilla in the for-IMRT plan. However, all these parameters did not impact mean dose in axillary level I area in the s-IMRT plan. It might be attributed to the better conformal dose distributions of the s-IMRT technique. Besides, Belkacemi et al [19] showed that the median dose delivered to axillary level I and II areas were higher in upper-quadrant tumor versus lower-quadrant tumor. When weighing optimal treatment patterns for individual patient with limited positive SLNs, not only the probability of residual non-sentinel lymph node disease but also these factors that impacted axillary dose distribution should be carefully estimated.

Though Z0011 and IBCSG 23-01 trials have presented encouraging results, patients in both trials had favorable diseases: 44% patients in Z0011 trial and all patients in IBCSG 23-01 trial had micro-metastases [3, 4]. Besides, the extent of radiation fields in Z0011 trial varied widely and a nontrivial minority of patients received extended nodal irradiation, including at least the supraclavicular and infraclavicular (axillary level III) lymph node [21]. Furthermore, another two trials—MA.20 and EORTC22922/10925 trials—demonstrated that regional lymph node irradiation could improve survival results in patients with positive axillary lymph nodes [22, 23]. In this study, no group of patients in both plans received a total dose of 45~50Gy, which has been usually believed to be the needed dose to treat the subclinical metastasis. Besides, the fraction dose was 1.1—1.2Gy but not a conventional fraction of 2Gy for axillary I area during whole breast irradiation. Therefore, for patients with limited positive SLNs and high risk of non-sentinel lymph node metastases, it is risky to rely on the incidental dose to axilla during whole breast irradiation to address the residual positive non-sentinel lymph nodes. In case of s-IMRT, it might be more risky since it delivered lower dose to axilla.

## CONCLUSION

When s-IMRT or for-IMRT plan delivered dose to axilla in patients with Whole breast irradiation, the s-IMRT plan reduced axillary level I area dose as compared with for-IMRT plan. For centers using s-IMRT technique, caution should be exercised when selecting to omit further axillary lymph node dissection for patients with breast conserving surgery and limited positive SLNs.

## MATERIALS AND METHODS

### Patients

Women with early stage breast cancer treated with breast conserving surgery and SLNB were included in this research. The main exclusion criteria were as follows: patients receiving further ALND after SLNB, receiving neo-chemotherapy or other pre-operative therapy. A total of 61 consecutive patients were collected and received CT scan followed by whole breast irradiation. This study was approved by the Ethical Committee and Institutional Review Board of the Fudan University Shanghai Cancer Center. All patients provided informed consent.

### CT scan

All patients were immobilized in a supine position on a breast tilt board (Med-Tech 350) with two arms fully abducted (90 degrees or greater) and externally rotated, head centered. A planning CT scan with a 5-mm interval from thyroid cartilage to costophrenic angle was obtained.

### Structure delineation

Breast tissue, axillary level I, II, III areas were delineated by the same physician (L Zhang) and reviewed by one experienced breast radiation oncologist (XL Yu). The breast clinical target volume (CTV) included the apparent CT glandular breast tissue. The breast CTV, axillary level I, II, III areas were delineated according to RTOG definitions [24]. The heart, lungs, spinal cord and contralateral breast were delineated as organs at risk.

### Treatment planning technique

All plans were done by the same senior physicist (LF Chen) using Pinnacle treatment planning software version 8.0 (Philips Medical, Madison, WI, USA). For each patient, two plans were generated: the s-IMRT plan and for-IMRT plan. The total prescribed dose was 50Gy in 25 fractions. A 10Gy/5Fx electron boost was added to tumor bed after whole breast radiation was finished.

### The s-IMRT plan

The s-IMRT was created with constraints on the maximum segment number, minimum MU/segment and minimum segment area. Five to seven beams were used to generate the s-IMRT plan. All plans were optimized to cover the breast planning target volume (PTV) and spare surrounding normal tissues as much as possible. The dose constraints for optimization were: 90% of the PTV received the prescription dose; 10Gy (V10Gy) to less than 30% of ipsilateral lung volume and 20Gy (V20Gy) to less than 20%; 10Gy (V10Gy) to less than 15% of the heart volume and 30Gy maximum dose to the heart and mean heart dose ≤6Gy for left-sided patients; maximum spinal cord ≤45Gy; contralateral breast mean dose ≤1.5Gy. For s-IMRT optimization, each plan was defined to have≤30 total segments, ≥10 MUs/segment and ≥10 cm^2^/segment..

### The for-IMRT plan

A field-in-field technique was used to manually generate the for-IMRT plan. The detail of the technique has been published [25]. Briefly, we used two tangential opposed beams for the affected breast with appropriate angles and MLC shapes with maximal ipsilateral lung sparing. Equal or almost equal weights were assigned to the two open fields, and the corresponding dose distribution was calculated and evaluated. To minimize the hot spot regions, subfields was copied directly from the original fields and the shape of each subfield was iteratively modified with aided visualization of 105% and 110% dose clouds in the beam's eye view. The number of subfields varied from 2 to 4. The percent volume of breast PTV receiving 50Gy should ≥90%, and maximum dose should not exceed 107% of the prescribed dose.

### Data collection

#### Dosimetric parameters

Following dosimetric parameters were collected: Dmean (mean dose), V47.5Gy, V45Gy, and V40Gy (percent volume receiving more than 47.5Gy, 45Gy and 40Gy) for axillary level I, II, III areas. Dmean, V30Gy, V20Gy (percent volume receiving more than 30Gy, 20Gy) for ipsilateral lung and Dmean for heart in left-sided patients.

#### Anatomic parameters

**Figure 2:**
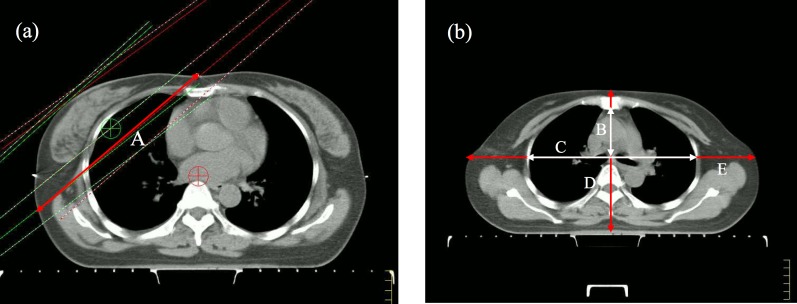
Definition of anatomic parameters: A = breast separation (red), B = thorax depth (white), C = thorax width (white), D = body depth (red), E = body width (red)

We collected body mass index (BMI) and breast CTV, breast shape, thorax index, body width, body depth on the simulation-localization-CT scans. The breast CTV was used as a surrogate for breast volume. The tangential fields-based breast separation was created to be a simple surrogate for breast shape. The breast separation was defined as the distance between the beam entrance points of medial and lateral tangential fields in the for-IMRT plan on the ISO plane. Thorax index was a parameter that reflected thorax shape, thorax index was defined as the chest width to depth ratio on the second rib inserted into the sternum plane. Chest depth was measured from the ventral surface of the vertebral body to the dorsal surface of the sternum on the midsternal line. Chest depth was measured between the inner surfaces of two symmetrical ribs, located perpendicularly to chest depth and divided in half at the widest point of the thoracic cage. Body width was defined as the distance between ventral and dorsal skin surface on the midsternal line. Body depth was defined as the distance between left and right skin surface on the extension of the chest depth line. The definitions of anatomic parameters are shown in Figure 2. The distance from upper border of tangential fields to humeral head was also measured.

### Statistical analysis

All statistical procedures were performed with SPSS version 13.0 (SPSS Company, Chicago, IL). Dosimetric parameters of axillary level I, II, III areas, heart, ipsilateral lung between the s-IMRT and for-IMRT plan were compared using the paired t test (two-sided). All anatomic parameters were cut off at the median value. Independent-sample t test was used to analyze potential factors that impacted dose distribution in axillary level I. Differences were regarded as statistically significant when *p* < 0.05.
